# Information foraging with an oracle

**DOI:** 10.1371/journal.pone.0295005

**Published:** 2023-12-28

**Authors:** Jeremy Gordon, Flavio Chierichetti, Alessandro Panconesi, Giovanni Pezzulo

**Affiliations:** 1 University of California, Berkeley, Berkeley, CA, United States of America; 2 Computer Science Department, Sapienza University of Rome, Rome, Italy; 3 Institute of Cognitive Sciences and Technologies, National Research Council, Rome, Italy; Roma Tre University: Universita degli Studi Roma Tre, ITALY

## Abstract

During ecological decisions, such as when foraging for food or selecting a weekend activity, we often have to balance the costs and benefits of exploiting known options versus exploring novel ones. Here, we ask how individuals address such cost-benefit tradeoffs during tasks in which we can either explore by ourselves or seek external advice from an *oracle* (e.g., a domain expert or recommendation system). To answer this question, we designed two studies in which participants chose between inquiring (at a cost) for expert advice from an oracle, or to search for options without guidance, under manipulations affecting the optimal choice. We found that participants showed a greater propensity to seek expert advice when it was instrumental to increase payoff (study A), and when it reduced choice uncertainty, above and beyond payoff maximization (study B). This latter result was especially apparent in participants with greater trait-level intolerance of uncertainty. Taken together, these results suggest that we seek expert advice for both economic goals (i.e., payoff maximization) and epistemic goals (i.e., uncertainty minimization) and that our decisions to ask or not ask for advice are sensitive to cost-benefit tradeoffs.

## Introduction

During decision-making, people and other living creatures have to constantly balance *exploration* and *exploitation*. The former refers to the selection of actions aiming to acquire task-relevant information, while the latter aims to secure a known reward. The diverse ways in which organisms address exploration-exploitation dilemmas have received significant attention in a variety of fields, from ethology to psychology, cognitive science and neuroscience [1–6]. The appearance of the exploration-exploitation dilemma across a wide array of biological and psychological domains hints at a common, fundamental challenge posed by the need to search, sometimes in vast multi-dimensional spaces, whether the target is a nutrient source, visual cue, memory, or behavioral policy [7]. Resolution of the dilemma typically comes in the form of heuristics and decision rules guiding a switch between modes, which are often adaptively influenced by the statistical properties of the environment and the cost dynamics of the task at hand. In ethology, where animal foraging behavior has been extensively studied, quantitative models such as the Marginal Value Theorem have been proposed. Results suggest that exploration (via patch switches) is prompted when a reward rate drops below a specific global threshold [8].

The multi-armed bandit paradigm, in which actions are met with an initially uncertain (and often stochastic) reward value, is another insightful mathematical framework to study the range of behavioral choices and strategies adopted when confronting exploration-exploitation trade-offs. For instance, recent work has explored the effect of spatial structure on large bandit tasks [9]. By learning a model of the environment, agents (using a function-learning model based on Gaussian Processes) and human participants could predict likely rewards at not yet visited locations to guide foraging decisions. Results showed that human participant strategies were consistent with an optimistic function learning approach, while also displaying a robust tendency to under-generalize the extent of spatial correlation. In a similar spatially structured bandit task, [10] find a relationship between cognitive load and the explore-exploit trade-off, with participants tending towards less exploratory behavior when memory demands are higher (manipulated by the presence of a memory aid tracking prior choice results). All the aforementioned studies of exploration-exploitation address situations in which exploration requires some form of search in the environment; for example, navigating a map to discover possible reward locations.

There is another, related kind of exploration-exploitation dilemma that has received less attention but that contemporary realities bring to the fore with a certain urgency: the case in which external advice is available to guide the search. For example, bank consultants offer advice about investment strategies, teachers offer advice to students [11], and industry expert consultants advise businesses. The advent of the web, however, has made such expert-advice-seeking experiences a part of daily life for billions of users seeking a diverse array of information types to aid decision-making. Web applications provide a variety of types of information signals, from simple user ratings to the responses of sophisticated algorithmic tools known as recommender systems, which (sometimes opaquely) make suggestions about entertainment to consume, restaurants to visit, and places to stay [12].

In this paper we use the term *oracle*, a term borrowed from the computer science literature, to refer to external advice of this sort, which is made available during information seeking. Because making use of such oracles often implies a cost (in terms of time, money, or both), deciding whether and how much to use such a resource as inputs to important important choices highlights an understudied variant of the exploration-exploitation trade-off. In cognitive science, there is a paucity of studies that address this trade-off explicitly, when oracle advice is available. One study explored the conditions under which participants are willing to pay to obtain expert advice, before making easy or difficult perceptual decisions [13]. Results show that participants are sensitive to both the utility and the reliability of information sources, and that they effectively select expert advice to address the most uncertain choices. However, this study only addresses simple (binary) perceptual decisions and it is unknown whether this finding generalizes to economic or preference-based decisions made in the real world where the (economic) costs of seeking expert advice as well as making the wrong decision can be compared more easily. Another study took a different approach and asked whether collaborative filtering methods widely used to build recommendation systems [12] or generalization models from cognitive science most resemble human recommendation behavior—specifically, the way humans select novel songs to complete a playlist [14]. In this work, a Bayesian generalization method from cognitive science [15] shows the strongest fit to human recommendation behavior and finds that human participants favour recommendations coming from the same Bayesian generalization model over other popular methods. While this study does not directly address exploration-exploitation, it at least shows that people are sensitive to the quality of the recommendation when making decisions about real-world tasks.

Two other studies have explored different facets of information sharing in the multi-armed bandit paradigm. In the first, a lab-based study probed the role of ambiguity aversion in bandit tasks by eliciting participants’ willingness to pay for authoritative information about an unknown bandit arm (in this case a true mean) [16]. In each of two treatments, a value is elicited about an unknown bandit arm: in a) the willingness to pay for true mean, and in b) the assessed value of the Gittins index, and these values are then compared. The Gittins index is defined as the sum of the expected value of an arm under current beliefs and its information value, via exploiting learnings from subsequent arm pulls, with an optimal MAB strategy involving choosing the arm with the present highest Gittins index [17].

Results show a lower than optimal Gittins index indicating higher willingness to pay for perfect information consistent with a suboptimal aversion to ambiguity. In the second study, a multi-agent algorithm is proposed to address a paradigm where teams of individuals can selectively share the results of individual arm pulls [18]. To solve this problem, agents must estimate the value of information received from the perspective of other team members. Though this work presents only an algorithmic analysis, findings show that a decentralized strategy can be developed which converges on the performance of a centralized strategy (endowed with perfect information). This finding demonstrates that selective information sharing can be an effective alternative to authoritative external information sources in the solution to explore-exploit tasks.

Finally, various reinforcement learning (RL) methods have shown that enhancing standard trial-and-error approaches to exploration with the ability to ask for external help or to consult external sources of knowledge is effective at addressing challenging navigation and problem solving tasks [19–22].

Taken together, these cognitive science studies suggest that people might be sensitive to a cost-benefit balance when asking expert advice, while the RL studies demonstrate that this advice is indeed effective in improving decision-making. Yet, this literature leaves two main questions unaddressed. First, we still lack a direct validation of the idea that people effectively balance the costs and benefits of relying on expert advice during economic or preference-based decisions, when its utility is uncertain and needs to be inferred. Second, it is currently unknown whether the benefits of consulting an oracle (in the sense defined above) should be linked exclusively to the economic side of the decision (i.e., as a way to maximize the decision payoff) or also to additional cognitive factors, such as the minimization of choice uncertainty, which have been consistently reported to influence choice behavior and information-seeking [23–25]. Answering this second question would help assess whether, when foraging for information with an oracle, people strive to maximize their utility, as postulated by traditional economic theories [26] or also strive to minimize their uncertainty (over and above maximizing economic payoff), as assumed by theories of bounded rational decision-making [27–30]. One aspect makes the study of oracle advice in the context of information foraging particularly intriguing. Oracle advice is rarely perfectly reliable. In conditions of suboptimal (error-prone) oracle information, what are optimal strategies, and how do they compare with human decision-making?

In the present work, we report results from two studies designed to address the above questions. Our studies aim to assess how participants face exploration-exploitation dilemmas, when advice from a costly, and possibly unreliable, oracle is available.

In Study A, we asked whether participants correctly recognize the conditions for which the benefits of consulting the oracle surpass its cost—that is, when making use of advice is the optimal information-seeking strategy. To this end, we systematically manipulated various contextual factors (i.e., reward distribution and expert reliability) that influence the value of expert advice, and measured whether participants adapt their choices to maximize payoff under these different contexts.

In Study B, we explored whether expert advice might have additional benefits beyond expected value. Specifically, we sought to measure individuals’ revealed preferences for uncertainty reduction (even in the absence of greater expected value), and analyzed links with attributes like intolerance of uncertainty (IUS) [31]. We designed a condition in which expert advice had no impact on average payoff, but reduced uncertainty by guiding participants to higher value locations.

From an information foraging perspective, studies A and B reflect the difference between one environment in which food sources are disposed randomly and another (more ecologically valid) environment in which they follow a structure, e.g., some areas are richer in nutrients than others, which foragers can learn through exploration [32–37]. Here, we add to this large body of literature the availability of an external information source—the oracle—that can be used in combination with or instead of standard exploration.

To preview our main results, Study A demonstrated that participants correctly recognize conditions for which seeking advice is optimal, despite showing a general trend towards under-use across conditions. In Study B, we found that many participants preferred to reduce uncertainty (payoff variance) using the expert, and that this trend was particularly strong for participants with higher IUS.

## Methods

### Experimental design

**Fig 1:**
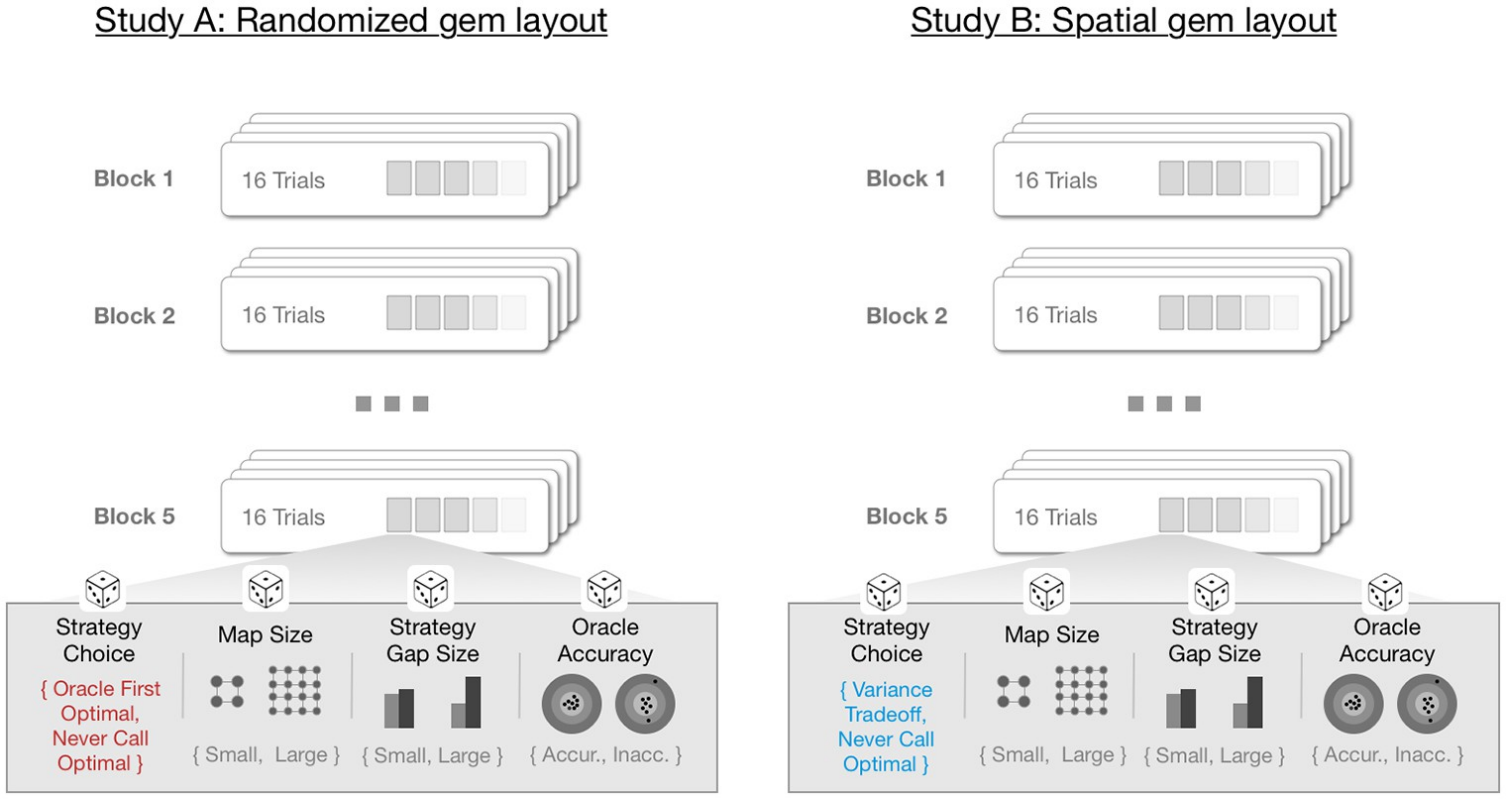
Experiment design. We performed two studies (Study A and B), both using a within subjects, 2x2x2x2 design. We manipulated strategy choice, map size, strategy gap size and oracle accuracy. The gem layout (randomized for Study A, and spatial for study B) affected only the strategy choice factor—that is, the effect of the two primary strategies: call oracle first, or never call—shown in red and blue text above. See the main text for details.

We report results from two studies (Study A and B), both using a within subjects, 2x2x2x2 design. The task in both studies involved completion of an online experiment administered on a laptop or desktop computer. Participants completed a series of 80 trials organized into 5 blocks, such that each block contained a balanced set of trials randomized according to each of the 4 conditions (see Fig 1). In each trial, participants moved an agent around a grid-like landscape, collecting up to 5 gems, each carrying (possibly unknown) reward value.

### Task & procedure

For both studies presented in this work, the task and procedure were the same except for the distribution of gem values (see descriptions of Study A and Study B below for details). The task design is detailed below.

**Fig 2:**
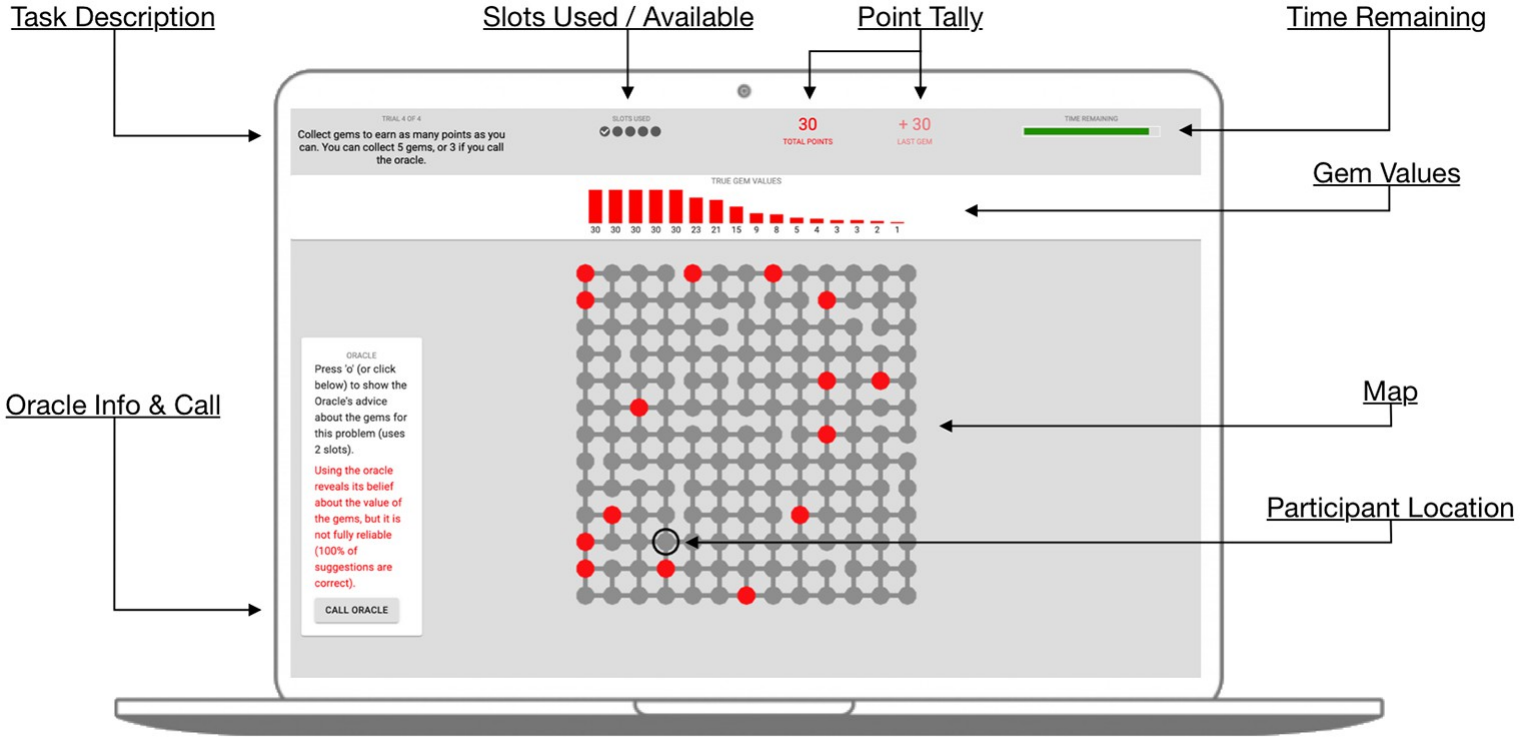
Task screen layout. See the main text for explanation.

Around each map, the screen layout showed a variety of information (See Fig 2). In the upper left corner the task instructions were continuously visible, which communicated that the participant should move around the map collecting gems to maximize total points. The slots remaining were shown next to this, indicating how many gems had been collected, and how many slots remained. Trials ended when all 5 slots had been filled, or when a 3-minute timer (in the upper right corner) expired. At the top of the screen was a visualization of the true gem value distribution. Each gem value was represented as a bar showing its value, along with a numerical representation. Participants used this information to decide how to complete each trial. These bars were shown in red for trials were the oracle was 100% accurate, and in blue for trials where the oracle was 75% accurate.

Participants navigated the map by using the arrow keys on the keyboard, and could visit any adjacent node that was connected by a line in the graph. To collect a gem, participants pressed the space bar. The gem’s value was shown and added to the total score after collecting each gem.

Additionally, an oracle was available to participants and could be called by clicking the “call oracle” button, or pressing ‘o’ on the keyboard. Calling the oracle revealed the oracle’s beliefs about where each gem value was located in the map, which was indicated by a numeric label showed on top of each gem’s location. Calling the oracle consumed 2 slots, hence the oracle cost, consistent across all trials and both studies, resulted from limiting the number of gems collected to 3 (as opposed to 5).

When a trial was finished (either by filling all slots, or the timer expiring), a dialog box appeared asking the participant to proceed to the next trial.

4 trials, an additional question was shown asking participants if they had the opportunity to solve the trial again, whether they’d make the same decision to call or not call the oracle.

After all 80 trials were finished, a post-task survey was shown composed of the shortened Intolerance of Uncertainty (IUS) Scale [31], followed by a unique participant ID, and the participants were thanked for their participation and told to close the browser.

Prior to starting, we randomized each participant into one of two studies.

#### Study A

In Study A, we aimed to explore whether people recognize the utility of seeking or not seeking expert advice—referred to in this experiment as the “oracle”—in addition to what other problem attributes affect this decision. Gems were randomly placed in the map, and each map varied according to four binary factors. *Map size* determined whether the map was small (9x9 grid) or large (13x13 grid). *Strategy choice* bucketed each map into those for which the optimal action is to call the oracle immediately (oracle first optimal, “OF”), and those for which the gem value distribution was such that the oracle benefit did not justify the cost, and therefore where never calling (“NC”) was optimal. *Strategy gap size* determined the size of the gap in expected value (and therefore the difficulty in discriminating the optimal strategy). Finally, *oracle accuracy* determined whether the oracle’s gem value rankings were 100% accurate (accurate condition), or 75% accurate (inaccurate condition)—oracle accuracy was made explicit to participants via the color of the oracle, and on-screen explanations. For implementation details on the strategy choice and strategy gap size factors, see Map Generation in the Methods section).

#### Study B

In Study B, we used the same set of four map factors, however, by introducing spatial structure to the distribution of rewards in the maps so that gems with similar values were closer to each other (similar to [9]), we manipulated the strategy choice participants needed to make. Specifically, the strategy choice condition assigned maps to either never call optimal (“NC”), consistent with Study A, or a new condition, variance trade-off (“VT”). In VT trials both strategies resulted in approximately equivalent expected value, but due to the availability of learnable statistical regularities in the maps, participants were able to make informed guesses about high-value gem locations, even without the oracle, resulting in higher variance (and therefore higher potential maximum value). Use of the oracle, on the other hand, reduced uncertainty by highlighting high value locations, but imposed the cost of the oracle, resulting in a similar expected value. In this second study, we hoped to understand how participants weighed this trade-off.

### Participants

Study participants were undergraduate students recruited by the authors at a university in Italy, in June and July 2022. 58 participants completed the online study. Participants had a mean age of 21.6 ± 2.4. 34 identified themselves as men, 13 as women, and 3 as other or declined to answer. All data was collected online. At the beginning of the experiment, participants gave anonymous consent and the authors had no access to information that could identify individual participants during or after data collection. The Ethical Committee of the National Research Council approved the study protocol.

### Map generation

Maps for the task were generated in a four step procedure: 1) generate and select gem value distribution, 2) define map connectivity, 3) distribute gems, 4) assign oracle beliefs. We detail each step below.

#### Generate and select gem value distribution

Our experimental design required us to select value distributions according to specific constraints related to optimal policy. Specifically, we needed to generate maps for which the optimal policy is to immediately call the oracle (oracle first or *OF*), and those for which the optimal policy is to not call the oracle and instead forage for gems without additional information (never call or *NC*). We designed a procedure to stochastically generate value distributions, by sampling from a clipped lognormal distribution in a specified parameter range, which we then stored into a pool of candidates. We then evaluated the expected value of each distribution assuming each of the two primary strategies, and computed the gap between the two, Δ, as: $\Delta =\frac{E_{OF}-E_{NC}}{E_{NC}}$. Value distributions with small and large gaps, balanced by map size, and optimal policy, were randomly selected for use in the study per our within subjects design. For details on the distribution sampling procedure, and expected value computations for each policy. See S1 File for additional details on value distribution and map generation.

#### Determine map connectivity

To ensure map navigation was non-trivial, we chose a grid-like map structure with some percentage of missing edges. For each pair of adjacent nodes, we flipped a p-coin to determine whether the nodes were connected. *p* = 0.9 produced maps with the desired level of variance and navigability. We additionally ensured that all nodes were reachable in the resultant graph.

#### Distribute gems

For each value distribution, and connectivity layout, we then generated two maps, one for each group of participants (random and spatial). For random maps, we placed each gem value, in randomized order, onto a free node in the graph. Only the 3x3 square of nodes adjacent to the starting position (middle coordinate) were disallowed for placement. For the spatially structured group, we used a procedure to generate patches making it more likely that higher value gems were placed near each other. To vary only the distribution of values and not other aspects of the map layout, we chose locations to place gems to be identical as those generated for the random maps, only the distribution of values within this group differed. We started by placing the highest value gem in a randomly chosen slot. Then, we proceeded in reverse value order. For each subsequent value, we flipped a p-coin to decide whether to place the gem in the next most proximal slot, or to randomly place it otherwise. Again, a value of *p* = 0.9 resulted in maps with clear spatial patches without being fully deterministic.

#### Oracle belief

Finally, to support trials where oracle information was inaccurate, we pre-assigned oracle beliefs for each map. Beliefs are a mapping of node locations to a value from the true gem value distribution. In accurate trials, beliefs match true gem values. In inaccurate trials, participants were told that 75% of oracle information was accurate. To achieve this, we randomly sampled 25% of belief values and shuffled these. In this way 75% of oracle beliefs were unaffected, and the remaining beliefs were guaranteed to be remapped to alternate values.

### Data structure

On each trial, we captured all relevant participant behavior including time of oracle request (if any), and trajectory of movements within the map, including the timestamp and whether any gem was collected. Definitions of each primary dependent variable (DV) are provided in Table 1.

**Table 1:** Dependent variable (DV) definitions.

DV	Definition
Oracle Requested	1 if oracle was requested on a given trial, 0 otherwise
Number of Moves	Count of moves (not including collecting gems) on a given trial
Performance	Score on trial divided by optimal score (assuming randomized map)

### Data analysis

Analyses for studies A and B were conducted with separate repeated-measures ANOVA, and linear regressions to investigate relations between variables.

### The algorithmic structure of map exploration with oracles

In this section we provide a mathematical analysis of Study A. Note that this analysis only applies to the case of 100% accurate oracles; however, it provides an upper bound to the value of any oracle and can be heuristically extended to the analysis of inaccurate oracles, by considering a potential loss of value that is proportional to their (in)accuracy.

Our aim is to describe and analyze some very natural exploration-exploitation strategies for our task and to show the existence of simple optimal strategies. Such strategies are also computationally friendly, in the sense that the value of the optimal outcome can be easily computed, thereby providing a useful benchmark to evaluate participants’ performance and behaviour. Although simple and intuitively appealing, these strategies exhibit a rather rich set of interesting properties.

We begin by recalling the task played by Study A’s participants. As a first step, the generic participant learns:

1. the multiset of (non-negative) values of the gems;
2. the location of the gems on the map, but not their values;
3. the number *t* of time units available, and the time penalty of an oracle call, denoted by *c*.

On the basis of this information, the participant can explore the map and, when a gem is reached, collect its value. The participant’s goal is to maximize the total value of collected gems. The participant can decide to consult the oracle at any time. When this happens, all gem values at their respective locations are revealed (recall that we are analyzing the case of perfect oracles only).

We assume that *t* and *c* are non-negative integers, since other choices are uninteresting. After learning *t*, *c*, and the multi-set of gem values, the participant starts exploring the map with the aim of collecting gems of maximum total value. The behaviour of the participant can be naturally subdivided into a sequence of *rounds*. In each round the participant performs an action that is relevant for the game: a gem can be collected (and its value learned and gained), or the oracle can be consulted (or “called”). Recall that, in Study A, the oracle—after being called—reveals the exact location and value of every gem present in the map yet to be collected. Note that the oracle can be consulted at any time during the game. In particular, it can be called *(i)* at the very outset, *(ii)* never, or *(iii)* in the course of map exploration after having collected one or more gems.

In particular, the participant can decide to call the oracle after having learned the values of some gems he or she has collected. We consider two classes of strategies: *adaptive* strategies, where the participant can decide if and when to call the oracle based on the values of the gems already collected, and *non-adaptive* strategies, where the participant does not use the value of the collected gems to determine if and when to call the oracle. Adaptive strategies can be more complex, since computing the next optimal action—as a function of what has been revealed so far—might require significant cognitive effort. In contrast, the pro’s and con’s of non-adaptive strategies are easier to assess. The dichotomy therefore captures an interesting computational aspect with plausible cognitive implications.

The goal of an “optimal” participant is to maximize his/her expected gain—that is, the total expected value of the collected gems. We speak of expected gain because the outcome is inherently probabilistic since the gems are distributed across locations uniformly at random.

Let us summarize and give a name to two simple non-adaptive strategies that a participant can employ:

- A participant can choose to begin the game by calling the oracle, and then collect gems in order of decreasing value in the remaining time—since calling the oracle has a time-cost of *c*, this strategy lets the participant collect the *t* − *c* gems of highest value. We called this strategy “Oracle First” (“OF”);
- A participant can also disregard, and never call, the oracle. This lets the participant collect a uniform-at-random subset of *t* gems. Note that the collected gems are uniform-at-random because of the absence of spatial conditioning in Study A—thus, whichever gem the participant chooses to collect next has a value that, from the point of view of the participant, is chosen uniformly at random among the values of the gems that are yet to be collected. This is the “Never Call” strategy (“NC”).

At first glance, one is tempted to conjecture that, depending on the distribution of gem values that the participant learns at the beginning of each game, one of the two OF and NC strategies might be optimal, *i.e.* it maximizes the expected gain. For instance, if all the gems have the same positive value, and calling the oracle has a positive cost *c* ≥ 1, then calling the oracle is necessarily suboptimal; in this case, NC achieves a larger gain than OF and it is, in fact, the only optimal strategy of the participant.

Conversely, suppose that there exists one gem of very high value and many gems of small value, and that the participant can either collect 2 gems, or call the oracle and collect 1 gem—that is, that *t* = 2 and *c* = 1. In such a setting, if the gap between the high value and the small value is large enough, a smart participant would call the oracle right away, and collect the high value gem (OF). To make things concrete, suppose that there are 9 gems of value 1 each, and 1 gem of value 10, and that *t* = 2 and *c* = 1. Here, the optimal strategy for the participant is to call the oracle right away to capture the one gem of value 10 (for an expected, and actual, gain of 10); the strategy NC collects two gems, whose values are determined uniformly at random, and results in the smaller expected gain of

$$\frac{9}{10}\cdot \frac{8}{9}\cdot \left(1+1\right)+\frac{1}{10}\cdot \frac{9}{9}\cdot \left(10+1\right)+\frac{9}{10}\cdot \frac{1}{9}\cdot \left(1+10\right)=1.6+1.1+1.1=3.8.$$

Thus, in this case, OF achieves an expected gain larger than that of NC, and it is the only optimal strategy.

Perhaps surprisingly, there are games where neither NC nor OF is optimal. As an example, consider a game with 3 gems of value 1 and 1 gem of value 5; also, suppose that *t* = 3 time units are available, and that *c* = 1. Then,

(i) NC results in an expected gain of $\frac{1}{4}\cdot (1+1+1)+\frac{3}{4}\cdot (5+1+1)=6$, since NC will collect the gem of value 5 with probability 3/4;
(ii) OF guarantees a gain of 5 + 1 = 6, since it will always collect the gem value 5, and exactly one gem of value 1.

Conversely, let us now consider the following adaptive strategy: the participant collects a gem as the first step; as observed, from the point of view of the player the value of this gem is sampled uniformly at random from the multi-set of available values. If this gem’s value is 5 (an event which happens with probability 14), the participant captures two other uniform-at-random gems, for a total gain of 5 + 1 + 1 = 7. If, instead, the first gem’s value is 1 (the complementary event, having probability 34), the participant calls the oracle in the second action and, in the third action, captures the gem of value 5, for a total gain of 6 = 5 + 1. The expected gain of this strategy is then

$$\frac{1}{4}\cdot 7+\frac{3}{4}\cdot 6=6.25>6.$$

In the above setting, the multiplicative gap between the gain induced by the overall best strategy, and the gain of the best of the two NC/OF strategies, is at least $\frac{6.25}{6}=\frac{25}{24}\approx 1.041666\ldots$. This simple example shows that a NC/OF-only participant can achieve no more than $\frac{24}{25}=96\%$ of the optimal gain.

In the “Adaptivity Gap” section, we study more complicated scenarios and show that NC/OF strategies can do significantly worse than 96% times the optimum. We conclude the section by asking a question that might have already tickled the reader’s mind: is there any particular reason why the NC and OF strategies were given such prominence in this paper besides their simplicity?

In the next section we show that, in fact, the best of the NC and OF strategy is *always* optimal in the class of non-adaptive strategies, a fact that justifies their intuitive appeal mathematically.

#### Optimal Non-adaptive strategies

As remarked, the OF and NC strategies are examples of non-adaptive strategies. The third strategy of the previous section, instead, is an example of an adaptive strategy: it calls the oracle depending on the random value of the first gem collected. More specifically it does so if, and only if, the first gem collected is not the one of highest value.

We now show that, given any distribution of gem values, at least one of NC and OF is optimal in terms of expected gain within the set of all possible non-adaptive strategies. Therefore, a participant that chooses to act non-adaptively can safely concentrate on those two strategies to find an optimal one—the set of non-adaptive strategies collapses on its two “extremes” at no cost for the participant.

The above result is formalized and proven in S1 File, Theorem 1, which we quote here for future reference:

*At least one of the NC and OF strategies is optimal in the set of non-adaptive strategies.*

A useful corollary of this fact is that it is possible to actually compute the expected gain of the best non-adaptive strategy, thus providing a useful benchmark to assess participant performance in the experiment. The formal derivation of this claim can be found after the proof of Theorem 1 in S1 File.

#### Optimal adaptive strategies

We have argued that an optimal non-adaptive strategy can always be found among the pair of strategies NC and OF—it is thus computationally easy to obtain an optimal non-adaptive strategy.

It turns out that it is also possible to compute the value of the best adaptive strategy. This can be done by resorting to the a well-known algorithm design technique: dynamic programming. The formal derivation can be found in S1 File.

#### The adaptivity gap

We have already argued that there exist settings where non-adaptivity makes the participant achieve no more than 96% of the optimal gain achievable by adaptive strategies. It is possible to come up with multi-sets of values for the gems such that non-adaptive strategies of oracle invocations can obtain no more than 91.73% of the optimal gain (attainable by adaptive strategies). The mathematical derivation can be found in S1 File.

#### Gem distribution generation procedure

In order to study participants’ responses to value distributions with different optimal strategy, and for which this strategy was easier or harder to detect (due to a larger or smaller gap between expected performance using each strategy), we designed a process to procedurally generate gem value distributions, and then evaluate the strategy attributes of each. Drawing value samples from the lognormal distribution produced the widest variety of non-trivial gem value sets, as well as sufficient diversity of optimal strategy to select the needed map configurations for our study.

$${\text{LogNormalPDF}}_{\mu ,\sigma^{2}}\left(x\right)=\frac{1}{x\sigma \sqrt{2\pi }}\text{exp}(-\frac{{\left(\text{ln}\left(x\right)-\mu \right)}^{2}}{2\sigma^{2}})$$

The initial pool was generated using parameter values of *μ* = 2, *σ* = 2.5. Samples were clipped to the range [1, 30] to avoid large value ranges that would be difficult to read from the bar-chart format used to visualize values in the task.

An initial pool of 1000 value distributions were generated, and each was evaluated to determinate its expected value under each of the two primary behavioral policies: oracle first, and never call.

As discussed in the Study B results, the expected value for never call in random maps (which is equivalent to random sampling) is simply the expectation of gem values times the number of slots, that is: $E_{NC}=t\cdot \frac{\sum_{i=1}^{n}X_{i}}{n}$.

The expected value for the oracle first strategy is similarly trivial: $E_{OF}$ is the sum of the values of the *t* − *c* gems of largest value. Here, *t* − *c* is the number of slots remaining after the oracle is called (that is, 3 in the reported studies).

## Results

### Study A results

In Study A, we aimed to explore whether people recognize the utility of calling (or not calling) the oracle, in addition to what other problem attributes affect this decision.

#### Sensitivity to the optimal strategy

In general, we found participants were sensitive to the utility of calling or not calling the oracle, despite not being specifically cued about strategy optimality. This is evident from the fact that their strategy differs dramatically between between the *NC* and *OF* conditions. Additionally, we found that in the vast majority of trials in which participants called the oracle, they did it when it was optimal; namely, before any gems are collected. Calls after 1 or more gems were collected (which should be considered suboptimal) were extremely rare.

**Fig 3:**
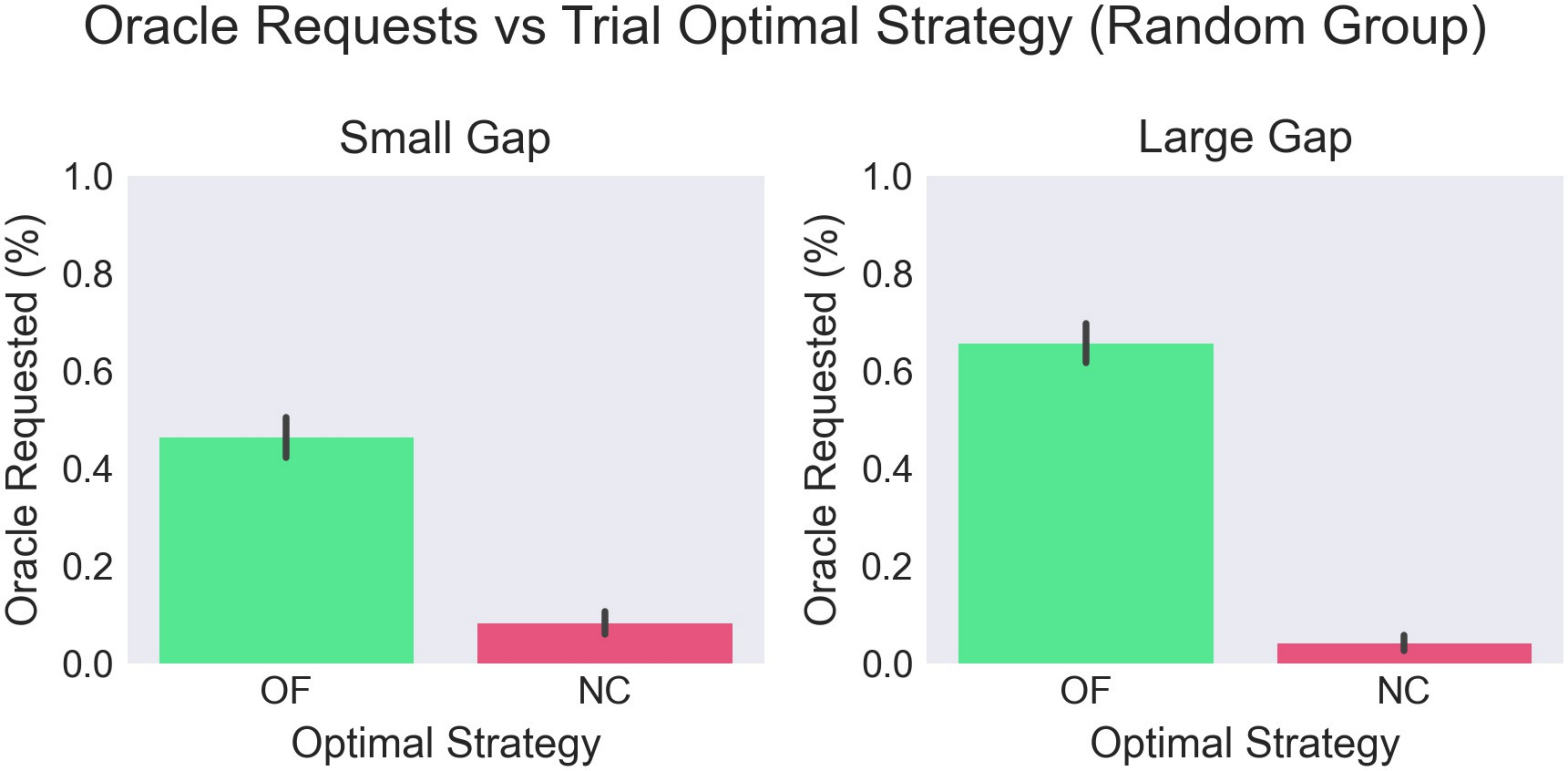
Oracle (under)-use against optimal strategy. Use was very low on NC trials, but only 55.8% of participants correctly used the oracle on OF trials. An interaction effect is seen showing that larger gaps were associated with increased use of the oracle on OF trials ($F(1,27)=35.9,p<.001,\eta_{p}^{2}=.066$).

**Fig 4:**
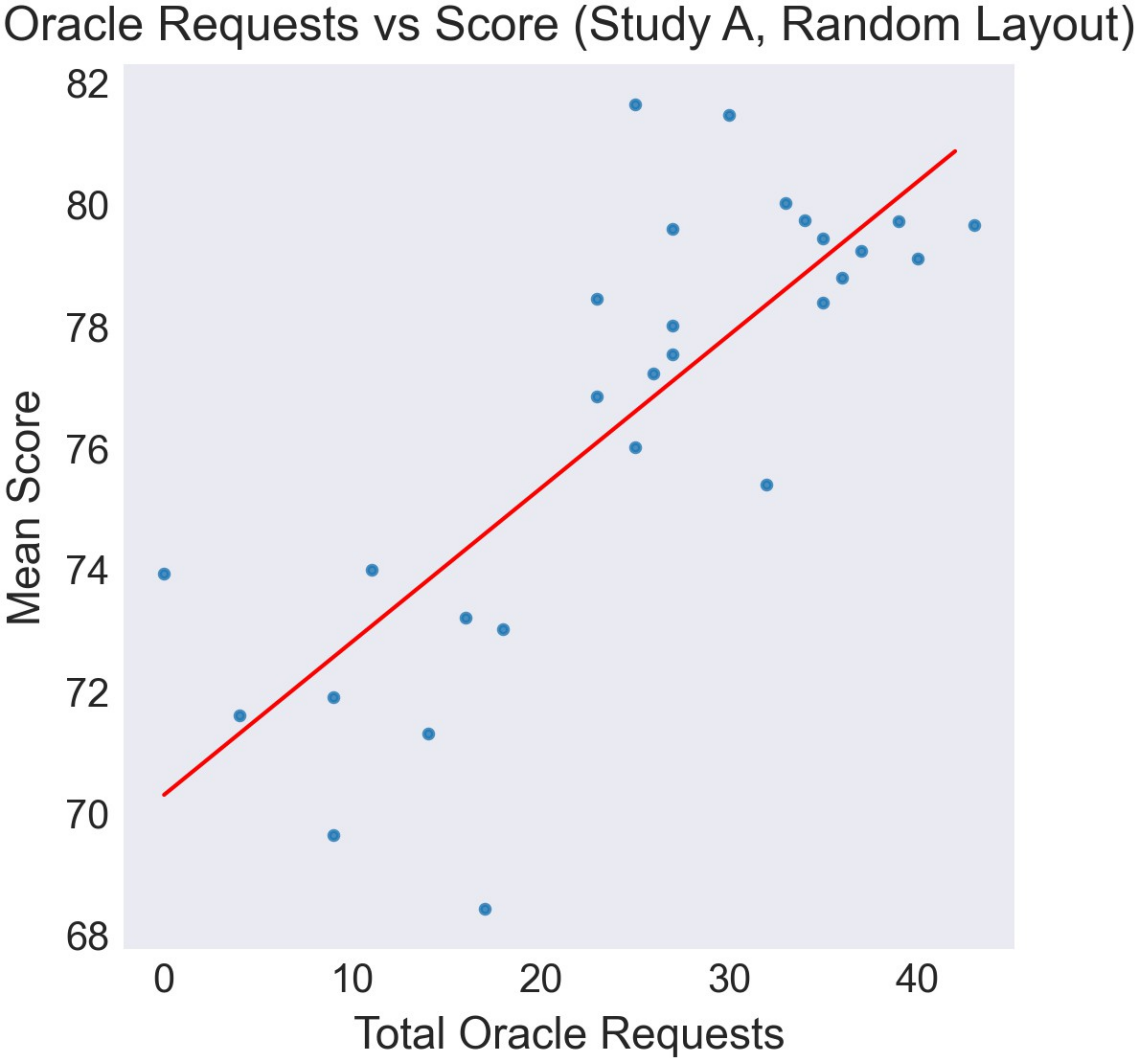
Scatter plots of oracle requests vs mean score across participants. Mean score is defined as the mean of total points collected by a participant across all non-practice trials. Total Oracle Requests is the count of trials where the participant called the Oracle, across all non-practice trials. A significant positive correlation is seen indicating the relationship between correctly identifying problems where the oracle is useful, and overall performance. Correlation is significant with Pearson-*r* = 0.78, *p* < 0.0001.

However, we find an asymmetry in oracle use between the two conditions. In the *NC* condition, over 90% of subjects correctly avoid using the oracle (and higher still when the gap size was large, such that the optimal strategy was easier to determine). Rather, in the *OF* condition between 30–55% of participants failed to request the oracle. In this condition, we see significant underuse of the oracle in maps where it would have supported improved performance, even when the gap size was large (see Fig 3). In line with this finding, we see a very strong positive trend in oracle use against score, highlighting that sufficient oracle use was critical to performance (see Fig 4).

#### Strategy satisfaction & regret

**Fig 5:**
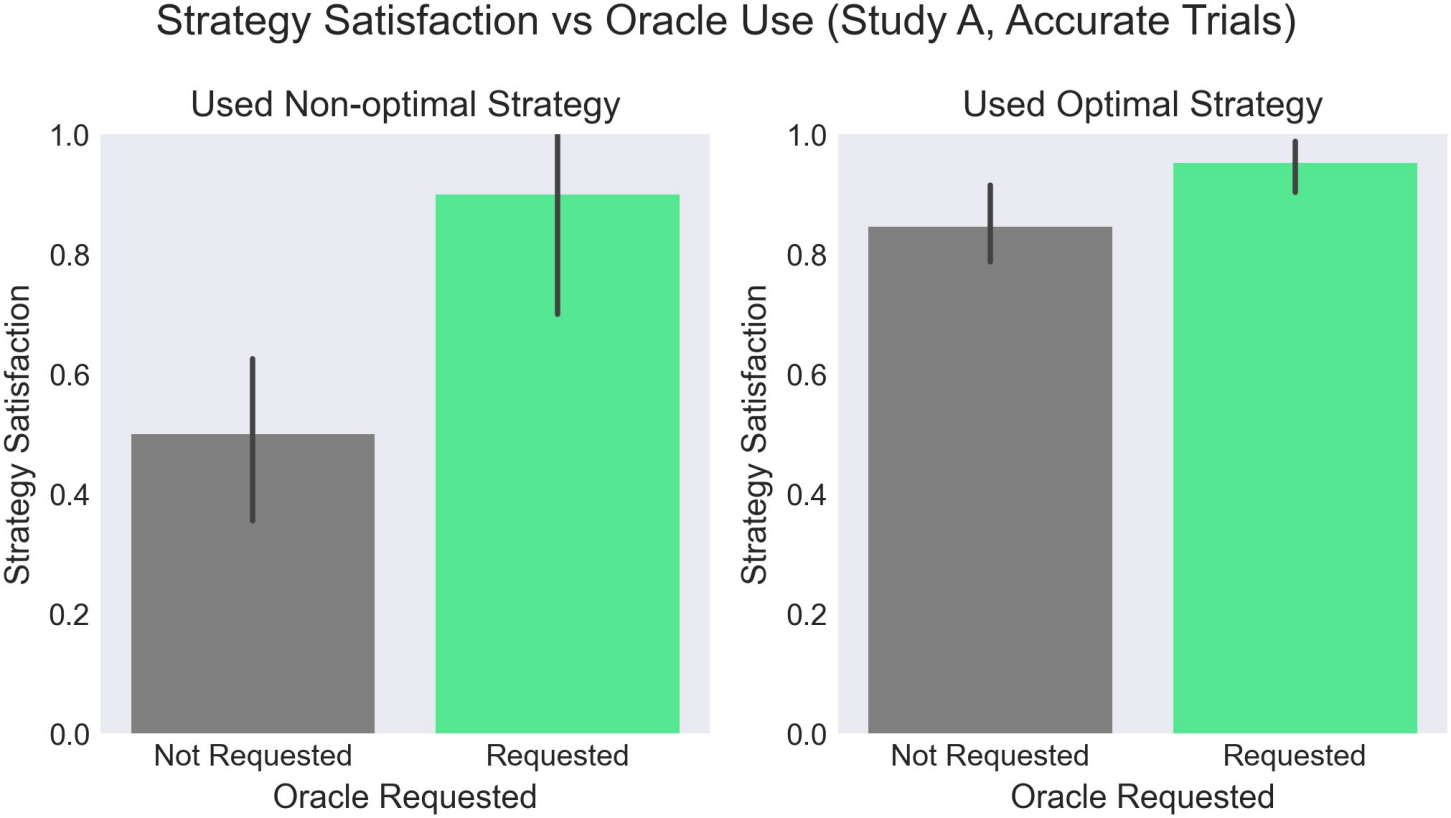
Strategy satisfaction, as measured by percent of participants responding yes to question: “If you could retry the same problem again, would you use the same strategy to call the oracle [not call the oracle?]”, post-trial. Here we analyze trials where the oracle was accurate, to simplify analysis of optimal behavior. We find a main effect showing higher satisfaction when the oracle was used, whether optimally or non-optimally ($F(1,2)=21.7,p=.043,\eta_{g}^{2}=.684$). Interestingly, three quarters (74.5%) of participants said they’d keep the same strategy even when they used a non-optimal strategy.

A question asked intermittently during the experiment following a trial result provides insights about participants’ satisfaction or regret with their chosen strategy. Participants were aware they made the right choice when they did (95.2%), with slightly less confidence (84.6%) on trials where not calling was optimal. When participants used the incorrect strategy, they detected this most easily on *OF* trials (where they should have called the oracle, but didn’t). However, half of these participants (50.0%) still said they’d keep the same strategy. See Fig 5 for ANOVA results.

### Study B results

In Study B, we explored how participants responded to trials where the oracle could be used to reduce uncertainty without a reduction in expected score, and how they weighed this variance trade-off.

**Fig 6:**
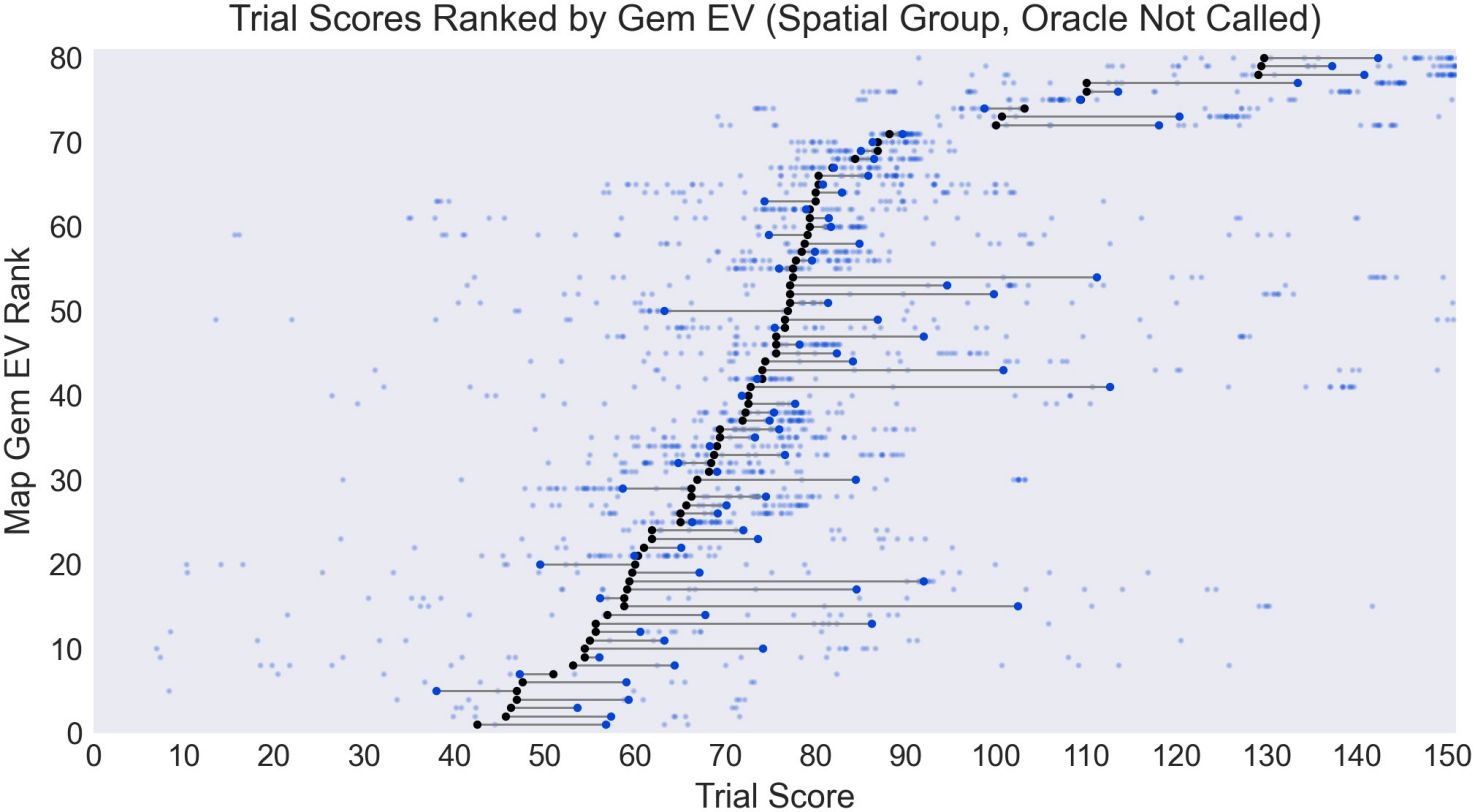
Comparison of trial score across participants who did not call the oracle (transparent blue points, with mean shown by opaque blue dot) versus trial’s expected score under a random policy (black dot). Mean performance was above expectation for most trials indicating that spatial information was exploited when participants chose not to call the oracle. Maps are ranked by expected score (“Gem EV Rank”).

The spatial structure added to all maps in Study B resulted in a noisy gradient of gem value centered around a randomly chosen location (for details see the Map Generation section in the Methods). To confirm participants detected this spatial regularity, we can compare scores with each map’s expected value under a random foraging policy, which is simply the gem value mean multiplied by the number of slots: $E_{NC}=t\cdot \frac{\sum_{i=1}^{n}X_{i}}{n}$. As shown in Fig 6, mean score (among participants not calling the oracle) was above the random policy expected value in the vast majority of trials, indicating clearly that spatial structure was exploited.

**Fig 7:**
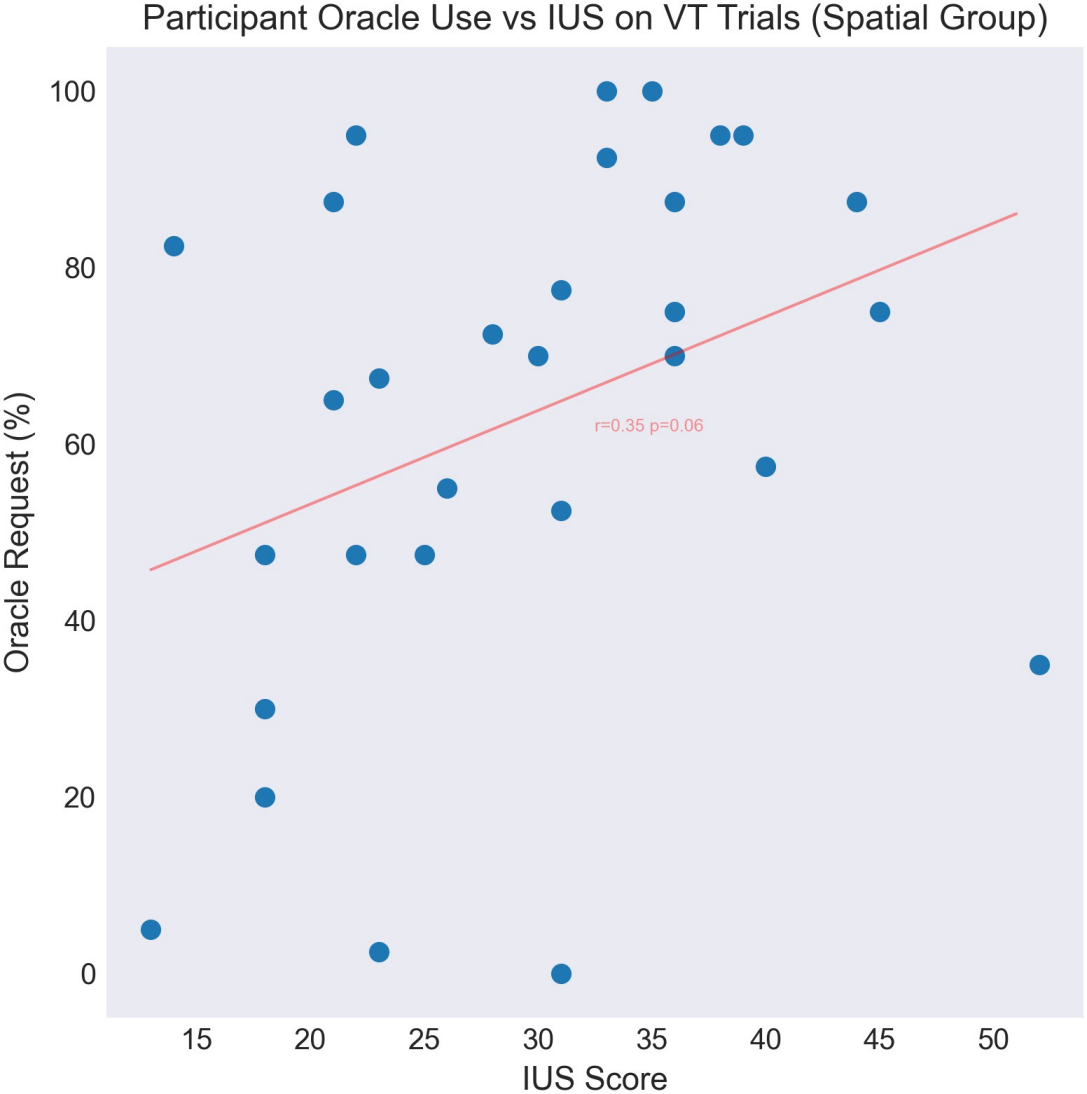
A positive correlation (*r* = 0.35, *p* = 0.06) is observed between participants’ IUS score and use of the oracle on VT trials.

**Fig 8:**
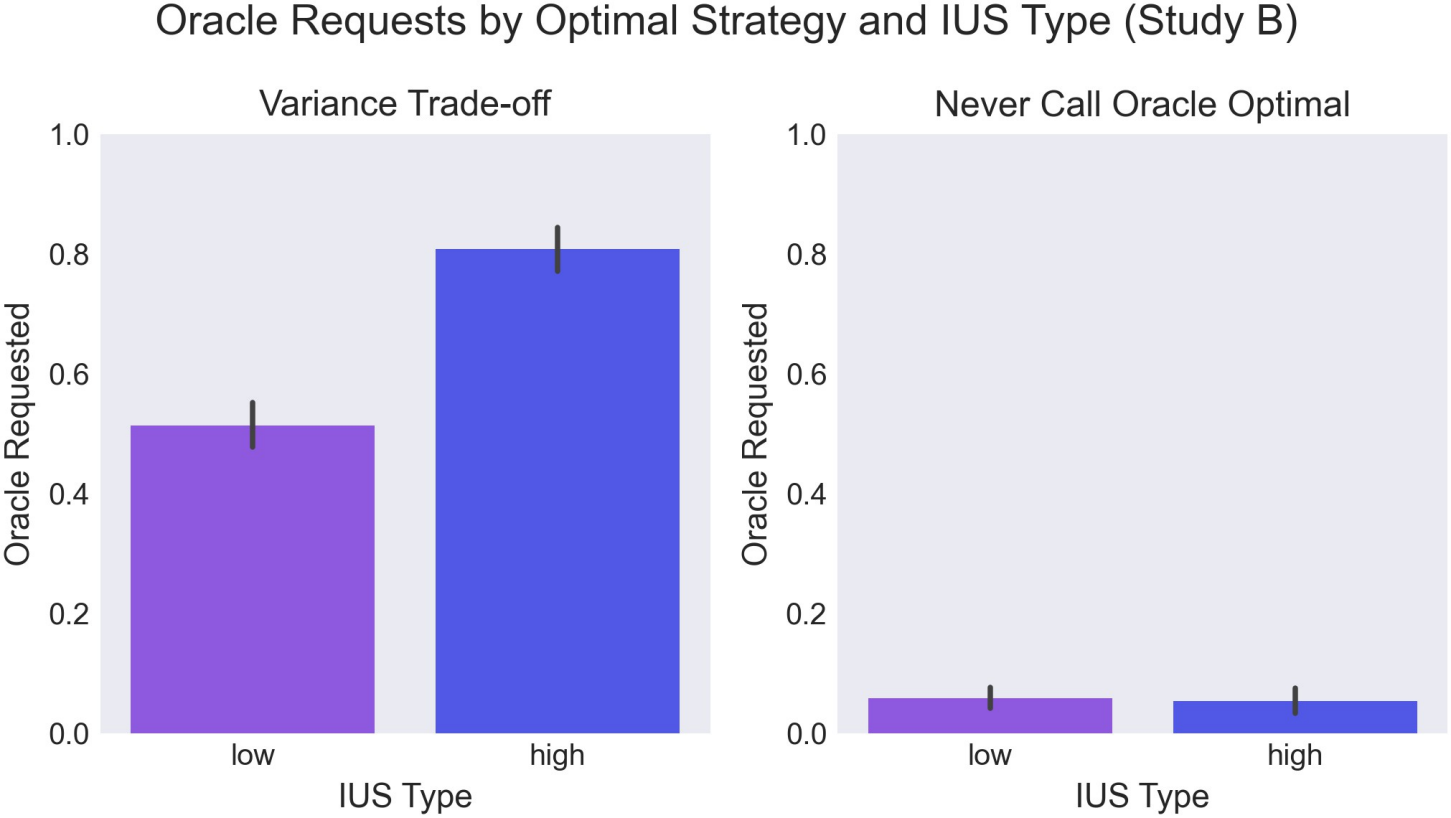
Use of the oracle vs IUS types (under or over median IUS score) in the spatial group (Study B). Left: among variance trade-off trials, right: among never call optimal trials. More requests were seen among high-IUS participants ($F(1,27)=6.1,p=.019,\eta_{p}^{2}=.115$), and in VT trials ($F(1,27)=158,p<.001,\eta_{p}^{2}=.714$). We additionally find an interaction between IUS-type and trial optimal strategy ($F(1,27)=9.2,p=.005,\eta_{p}^{2}=.127$), showing that the most oracle requests were seen among high-IUS participants in VT trials (*T* = 3.05, *p* = .005, *BF* = 8.6).

We then investigated participants’ handling of variance trade-off maps. Consistent with findings from Study A, participants used the oracle significantly more frequently on VT trials. However, substantial individual variation is observed, with some participants never using the oracle (and therefore opting into a gamble with higher risk but higher upside), and some using it in all trials (see Fig 7). We predicted that participants’ disposition towards uncertainty (as measured by the intolerance of uncertainty index) would correlate with this decision. Indeed, we find that high IUS participants were significantly more likely to call the oracle on VT trials (see Fig 8). This finding is consistent with high-IUS participants being especially motivated by the uncertainty reduction afforded by use of the oracle.

**Fig 9:**
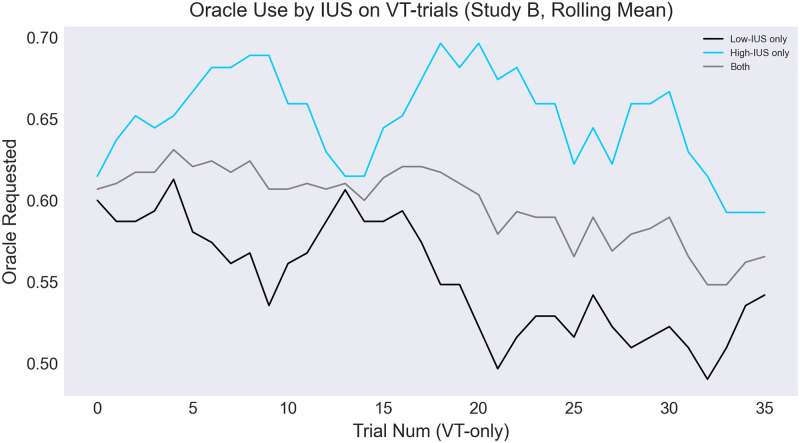
Oracle use trends through experiment (VT trials only, spatial group). Lines show a rolling average of oracle use across a 5-trial window, for high-IUS participants (blue line), low-IUS participants (black line), and across all participants (gray line).

Finally, when analyzing oracle use longitudinally through the experiment, we find a negative trend that is especially steep for low-IUS participants (see Fig 9).

## Discussion

In many real life situations, such as when choosing a movie to watch, a restaurant at which to dine, or a place to visit, we have the choice between exploiting our previous knowledge or exploring new alternative possibilities, either by searching in the environment or by seeking external advice. The dynamics of exploration via environmental search has received significant attention in cognitive science and neuroscience [1–3, 5, 6]. Comparatively, we know much less about the conditions that move people to ask for expert advice during decision-making when doing so has a cost.

Here, we designed two studies to investigate whether people correctly balance the costs and benefits of seeking expert advice during an economic decision (Study A) and whether they might seek external advice for other reasons, such as to lower choice uncertainty, even when this has no effect on average payoff (Study B).

In both studies, participants navigated a maze-like environment to collect a limited number of gems carrying different values. Gem positions were visible in the map, and the overall value distribution was shown, but the locations of each value were not initially available. For each trial, participants faced the choice between free foraging to find high value gems, or an “oracle” that showed the location of the gem values in the map—at the cost of collecting two less gems. By manipulating various choice parameters, such as the gem distributions, the reliability of the oracle and the statistics of gem placement (i.e., random placement versus a spatial structure in which gems with similar value were closer), we created conditions in which calling the oracle leads to a higher or lower payoff (Study A) or higher or lower payoff variance (Study B)—and we studied how this changed participants’ use of the oracle.

Analyses from Study A showed that participants are able to detect NC trials (via their gem value distribution) in which the cost of the oracle is not justified by the value. Participants are able, on average, to determine when use of the oracle is valuable as well, as seen from the strong preference for calling the oracle in OF trials when this is optimal, and that additionally, participants rarely call the oracle after the first move (when it is never optimal). However, we found a general trend of oracle under-use across conditions. A possible explanation for this asymmetry in our results is that it was more difficult to assess the benefit of the oracle in OF trials. Indeed, while participants could learn about task dynamics and tune their strategy across the experiment, direct feedback about strategy use was not available (score was provided, but without information about optimal score).

An asymmetry is also seen in particpants’ satisfaction with the decision to call or not call the oracle. Satisfaction was lower after trials in which the non-optimal strategy was chosen (as expected), and especially low when the oracle was not called (only 50% of participants said they would make the same choice again after not calling, compared with 90% for those who called the oracle). This latter result might be explained by the risk inherent to not calling the oracle—if a participant is unlucky with their randomly collected gems, they may be especially likely to regret their decision. In contrast, calling the oracle implies a simple policy (collect the oracle’s highest-valued gems) that is unlikely to result in surprises (especially in trials where the oracle is accurate).

In sum, the results of Study A align well with previous findings that participants are sensitive to the costs and benefits of asking for costly expert advice before a decision [13]—although they show some oracle under-use—and indicate that this sensitivity is apparent during economic decisions that imply payoff maximization. Interestingly, participants were able to infer the utility of the oracle from not only reliability information (which was stated explicitly) but also from the gem distribution, which provides a much more indirect indication.

In Study B, we found that participants prefer calling the oracle even when there is no payoff benefit—because the task includes spatial regularities that can be exploited to infer the best gem locations. Our results make it clear that this is not explained by participants being unable to understand the spatial structure, since it was effectively exploited in trials where the oracle was not called. Instead, this result suggests that participants were motivated by a drive to reduce their outcome-level uncertainty (about the variance of their payoff) and policy-level uncertainty (about where to move next), above and beyond payoff maximization. Supporting this idea, we found a positive relationship between participants’ Intolerance of Uncertainty (IUS) score, and use of the oracle on variance trade-off trials (see Fig 7). This finding could be explained by the fact that the spatially structured maps in Study B induce an enduring policy-level uncertainty—even after a high-value gem is collected, it is not clear which direction to forage in next. And yet, the spatial regularities, once detected, imply that there is in fact a pattern, which there is often (at least initially) insufficient information to fully deduce. If so, it is reasonable to expect this ambiguity to affect high IUS participants most significantly, and encourage use of the oracle as the only recourse to reduce this expected uncertainty.

Taken together, the two studies show that peoples’ preference for expert (oracle) advice is evident both when it conveys economic value (i.e., increases economic payoff) as well as epistemic value (i.e., it reduces payoff variance and choice uncertainty). Both reward maximization and uncertainty reduction are key determinants of choice in theories of bounded rational decision-making that take information costs into consideration [27–30, 38] and here we show that they can be in play (simultaneously) even during exploration-exploitation scenarios that involve external sources of evidence. Note that the first experiment shows a general under-use of expert advice across conditions, whereas the second experiment shows a general preference for expert advice in ambiguous conditions. While these results may be seen to be in tension, another interpretation is that the under-use in study A highlights a tolerance or even affinity for the unguided gamble which also minimizes the cognitive load of navigating—in a landscape with no structure, while values are uncertain, there is no right or wrong navigation strategy. As previously mentioned, study B poses a different type of ambiguity: the learned awareness of spatial structure confers an irreducible uncertainty that requires ongoing, and rather challenging, pattern identification and control. This interpretation is supported by the general increase in gambles (seen as reduced oracle use) through the experiment on variance trade-off trials, as participants improve their understanding of the spatial structure.

The results of our studies have both theoretical and practical implications for the study of information foraging in the real world. From a theoretical perspective, our results show that people are sensitive to the costs and benefits of obtaining external advice during decision-making—and that the benefits of external advice can go beyond economic payoff. From a more practical perspective, the results of this study speak to the usefulness of requesting (or not requesting) expert advice during real-world decisions, such as when consulting recommendation systems or social networks. While our study addresses a simplified choice situation, its methodology could potentially be reused (and extended) in future investigations of how people seek expert advice when deciding about movies, restaurants, or travel destinations.

This study has various limitations that would need to be addressed in future studies. First is a further exploration around a puzzling aspect of our findings. Namely, in both study A and B, we find slightly increased use of the oracle in small maps, compared with large maps—despite the expectation that larger maps pose greater outcome- and policy-level uncertainty.

An additional limitation in this work is that for simplicity, we addressed the case of standard economic decisions in which the ranking of participants’ preferences is clear (i.e., the task was designed such that all participants would prefer gems with greater value). However, expert advice, such as recommender systems, are often used in choice situations in which the ranking of preferences is less clear, for example when finding music or restaurants online. Indeed, one of the main goals of recommender systems is to infer the preference ranking of individuals from data [12]. Apart from the difficulty of inferring preferences, what makes these scenarios more subtle is the possibility that the preferences could be influenced by the advice received or (retrospectively) by the choice itself, rather than being fixed [39, 40]. Understanding how people manage the costs and benefits of seeking expert advice in those more subtle, real-life conditions remains an open challenge.

## Conclusion

In this work, we presented two studies exploring information foraging behavior when individuals have access to expert (but costly) advice in the form of an oracle. Our results show that peoples’ preference for this advice is evident both when it conveys economic value (i.e., increases economic payoff) as well as epistemic value (i.e., it reduces payoff variance and choice uncertainty). We show that both reward maximization and uncertainty reduction can be in play (simultaneously) even during exploration-exploitation scenarios that involve external sources of evidence. The simplified choice situation explored in this work highlights a more generic methodology which may be used and extended in future investigations of how, and when, people seek expert advice in real-world contexts.

## Supporting information

S1 File An algorithmic analysis of Study A: Proofs. In this Section we provide rigorous mathematical proofs for various claims made in the main text.
(PDF)
