# Supplementary material for: Information foraging with an oracle
Source: PLoS One. 2023 Dec 28;18(12):e0295005. doi: 10.1371/journal.pone.0295005 (PMC10754449; doi:10.1371/journal.pone.0295005)
Supplement: S1 File — In this Section we provide rigorous mathematical proofs for various claims made in the main text. (PDF) [file pone.0295005.s001.pdf]

Supporting Information: Information Foraging with an Oracle

Jeremy Gordon[1], Flavio Chierichetti[2], Alessandro Panconesi[2◑], Giovanni Pezzulo[3*◑],

1 University of California, Berkeley, Berkeley, CA, United States
2 Computer Science Department, Sapienza University of Rome, Italy
3 Institute of Cognitive Sciences and Technologies, National Research Council, Rome, Italy

◑These authors contributed equally to this work.
* giovanni.pezzulo@istc.cnr.it

An Algorithmic Analysis of Study A: proofs

In this Section we provide rigorous mathematical proofs for various claims made in the main text.

The best non-adaptive strategy

We now show that — given any distribution of gem values — at least one of the NC and the OF strategies is optimal (in terms of its expected gain) in the set of non-adaptive strategies.

Theorem 1. *In a generic game, at least one of the NC and OF strategies is optimal in the set of* non-adaptive *strategies.*

Proof. First of all, we prove that the exists an optimal non-adaptive strategy that is deterministic (i.e., non-randomized). Indeed, take an optimal non-adaptive strategy σ. Since σ could be random, a run of σ will either avoids calling the oracle, or calls it in the first round, or in the second round, ..., or in round t. Let p_∞ be the probability that σ avoids calling the oracle and, for $i = 1, \ldots, t$, let p_i be the probability that σ calls the oracle in round i. Then, if $\mathbb{E}_i$ is the expected gain conditioned on σ calling the oracle in round i, and if $\mathbb{E}_\infty$ is the expected gain conditioned on σ not calling the oracle, we have that the expected gain of σ is

$$\mathbb{E}_\sigma = \sum_{i \in \{1,2,\ldots,t,\infty\}} (p_i \cdot \mathbb{E}_i) \, .$$

Let $i^\star \in \{1, \ldots, t, \infty\}$ be such that $\mathbb{E}_{i^\star} \geq \mathbb{E}_i$ for each $i \in \{1, \ldots, t, \infty\}$. Observe that there might be multiple $i^\star$'s with this property. Then, the strategy that deterministically calls the oracle in round $i^\star$ — or, if $i^\star = \infty$, the NC strategy — has an expected gain of $\mathbb{E}_{i^\star}$ which, by $\mathbb{E}_{i^\star} \geq \mathbb{E}_i$ for each i, and by $p_1, \ldots, p_t, p_\infty$ being a probability distribution, entails that $\mathbb{E}_{i^\star} \geq \mathbb{E}_\sigma$. Thus, we have created a deterministic non-adaptive strategy that is no worse than the chosen (optimal) randomized non-adaptive strategy.

We then assume that there exists an optimal non-adaptive strategy in the set $S = \{\sigma_1, \ldots, \sigma_t, \sigma_\infty\}$ — i.e., the set that contains, for $i \in \{1, \ldots, t\}$, the deterministic strategy that calls the oracle in round i (σ_i), as well as the NC strategy (σ_∞). Observe that σ_1 is the OF strategy.

Recall that, for $i \in [n]$, we let $\mathbb{E}_i$ be the expected gain of the σ_i strategy, and we let $\mathbb{E}_\infty$ be the expected gain of σ_∞. (Then, $\mathbb{E}_{OF} = \mathbb{E}_1$ and $\mathbb{E}_{NC} = \mathbb{E}_\infty$). We now show that

$\max(\mathbb{E}_1, \mathbb{E}_\infty) \geq \mathbb{E}_i$ for each $i \in \{1, \ldots, t, \infty\}$ — this will prove our main assertion. We consider two cases:

- first, consider the strategy σ_i, for $i \in \{2, 3 \ldots, t-c\}$. This strategy, in its first $i-1$ rounds, picks a uniform-at-random subset A of gems conditioned on $|A| = i-1$, and, in its last $t-c-i+1$ rounds picks $t-c-i+1$ gems of maximum value (among the remaining ones). In particular, then, strategy σ_i picks a subset of $t-c$ gems; given that strategy σ_1 picks a subset of $t-c$ gems of maximum total value out of the full set of gems, it must be that $\mathbb{E}_i \leq \mathbb{E}_1$. It follows that, for each $i \in \{2, 3, \ldots, t-c\}$, σ_i is no better than σ_1.

- Now, consider the strategy σ_i, for $i \in \{t-c+1, t-c+2, \ldots, t\}$. The strategy σ_i is unable to collect any gem after its call to the oracle, since the oracle requires c time units, and only t time units are available. Thus, σ_i will only collect $i-1$ uniform-at-random gems. Recall that the strategy σ_∞ collects t uniform-at-random gems. Since $i-1 \leq t-1 < t$, and since no gem has negative value, it must hold that $\mathbb{E}_i \leq \mathbb{E}_\infty$. Thus, for each $i \in \{t-c+1, t-c+2, \ldots, t\}$, σ_i is no better than σ_∞. If gems of negative value are present, this property might fail to hold — e.g., if each gem has value -1, σ_∞ has an expected gain of $-t$ whereas each other σ_i has an expected gain of at least $-t+1 > -t$.

It follows that (at least) one of σ_1 and σ_∞ (that is, of OF and NC) is an optimal strategy of S and, as a consequence, it is an optimal non-adaptive strategy. □

Observe that the expected gain of the NC strategy is equal to t times the average gem value, $\mathbb{E}_{NC} = \mathbb{E}_\infty = t \cdot \frac{\sum_i X_i}{n}$. And, the expected gain of the OF strategy, $\mathbb{E}_{OF} = \mathbb{E}_1$, is equal to the total value of the $t-c$ gems of highest value.

These two observations, together with Theorem 1, make it possible to efficiently compute the expected gain of the optimal non-adaptive strategy: an algorithm could just return the largest of $\mathbb{E}_1$ and $\mathbb{E}_\infty$.

The best adaptive strategy

We now give an algorithm, based on the well-known dynamic programming technique, for computing an optimal adaptive strategy.

Let S be a multiset of gem values; imagine S to be the multiset of values of the gems that are still on the map, after some number of moves. If, at some point in the past, the participant has called the oracle, the optimal move for S is easy to compute: the participant should collect one of the gems of highest value among those in S. Otherwise, the participant should decide whether to call the oracle in the next move, or whether to collect a uniform-at-random gem.

Let us define $O_{t,c,S}$ to be the maximum expected gain that an adaptive strategy can achieve (i) with t time units available, (ii) with an oracle cost of c, (iii) on a map containing gems whose multiset of values is S.

Clearly if $t \leq 0$, or if $S = \varnothing$, then $O_{t,c,S} = 0$: if there are no moves, or no gems, available, then the participant cannot collect any gem, and ends up with a null total gain. Otherwise, we have that

$$O_{t,c,S} = \max\left(\operatorname{avg}_{v \in S}\left(v + O_{t-1,c,S-\{v\}}\right), T_{t-c}(S)\right),$$

where $T_{t-c}(S)$ is the total value of the $t-c$ gems in S of highest value, or the total value of the gems in S if $|S| < t-c$ (and, $T_k(S) = 0$ if $k \leq 0$).

The recurrence chooses the best of the two options available to the participant: either collect a uniform-at-random gem (and obtain the value v of that gem plus the optimal gain that can be achieved with $t-1$ units of time on a map with gem values

$S - \{v\}$), or call the oracle (and obtain the total value $T_{t-c}(S)$ of the $t - c$ gems of highest value in S). Clearly, the optimal strategy would select an option that results in the largest expected value.

Thus, we have expressed the value of $O_{t,c,S}$ for the base cases ($S = \varnothing$, or $t \leq 0$), and given a recurrence for the other cases. Unwinding the recurrence gives rise to a dynamic program that computes the maximum expected gain achievable by an adaptive strategy.

It is easy to observe that, for a set of n gems, this dynamic program runs in time $O(n \cdot 2^n)$. In fact, if there are only k distinct gem values $v_1, \ldots, v_k$, and if the generic value v_i is shared by g_i gems, we have that $n = \sum_{i=1}^{k} g_i$ and that the dynamic program can also be implemented to run in time $O(k \cdot n^k)$ — that is, if there are constantly many distinct gem values, the runtime becomes polynomial.

The Adaptivity Gap

We have already argued that there exist settings where non-adaptivity makes the participant achieve no more than 96% of the optimal gain achievable by adaptive strategies. In this section we show that the gap can be larger: there are settings where non-adaptive users can collect no more than 91.73% of the optimal gain.

Let $\phi = \frac{1+\sqrt{5}}{2} \approx 1.61803\ldots$ be the Golden ratio, and recall that $\frac{1}{\phi} = \phi - 1$. We define a Golden ratio-based game which will allow us to give a stronger bound on the adaptivity gap:

Definition 2 (Golden Game)**.** *For a large enough n, let the map be composed of $g_1 = n - 1$ gems of value $v_1 = 1$, and $g_2 = 1$ gem of value $v_2 = \frac{n}{\phi}$; the player has $t = \left\lceil \frac{n}{\phi} \right\rceil$ time units available, and calling the oracle requires $c = \left\lceil \left(\frac{2}{\phi} - 1\right) \cdot n \right\rceil$ time units.*

In particular, observe that v_2 and t are approximately equal to $0.61803 \ldots \cdot n$, and that $c \approx 0.23606 \ldots \cdot n$.

We begin our analysis by computing the expected gain of the two extremal non-adaptive strategies on this game.

Lemma 3. *Both the NC, and the OF, strategies induce an expected gain of $n \pm O(1)$ on the game of Definition 2.*

Proof. The NC strategy captures the gem of value v_2 with probability $\frac{t}{n}$, and captures either $t - 1$, or t, gems of value v_1: its expected gain is then

$$\begin{aligned} \frac{t}{n} \cdot v_2 + (t \pm O(1)) \cdot v_1 &= \frac{\left\lceil \frac{n}{\phi} \right\rceil}{n} \cdot \frac{n}{\phi} + \frac{n}{\phi} \cdot 1 \pm O(1) = \frac{n}{\phi^2} + \frac{n}{\phi} \pm O(1) \\ &= \frac{n \cdot (\phi - 1)}{\phi} + \frac{n}{\phi} \pm O(1) = \frac{n \cdot \phi}{\phi} \pm O(1) = n \pm O(1). \end{aligned}$$

The OF strategy, instead, captures the gem of value v_2 and $t - c - 1$ gems of value v_1, with probability 1. Its total gain is then

$$\begin{aligned} v_2 + (t - c - 1) \cdot v_1 &= \frac{n}{\phi} + \left(\frac{n}{\phi} - \left(\frac{2}{\phi} - 1\right) \cdot n\right) \cdot 1 \pm O(1) \\ &= \frac{n}{\phi} + \left(1 - \frac{1}{\phi}\right) \cdot n \pm O(1) = n \pm O(1). \end{aligned}$$ □

We can now bound the expected gain of optimal non-adaptive strategies.

Corollary 4. *The expected gain of optimal non-adaptive strategies for the game of Definition 2 is* $n \pm O(1)$.

Proof. Apply Theorem 1 and Lemma 3. □

We now show that there exists an adaptive strategy with an expected gain of $1.09016\ldots \cdot n$ — roughly, 9% more than the expected gain of optimal non-adaptive strategies.

Theorem 5. *There exists an adaptive strategy for the game of Definition 2 with expected gain* $(5 \cdot \phi - 7) \cdot n \pm O(1)$.

Proof. Consider the following adaptive strategy: in the first phase of the game, the participant captures $t - c - 1$ uniform-at-random gems. If the participant captures the high-value gem (i.e., that of value v_2) during the first phase, the participant will keep capturing uniform-at-random gems for the remainder of the game. Otherwise, right after the end of the first phase, the participant will (i) call the oracle, and (ii) capture the high-value gem, just before the game ends.

Observe that the behavior of the participant is determined by the event $\xi =$ "the high-value gem is captured during the first phase". The probability of ξ is equal to $\frac{t-c-1}{n}$ (i.e., the probability of drawing a red ball in $t - c - 1$ draws without replacement from an urn containing one red ball, and $n - 1$ blue balls). If ξ happens, the participant captures the gem of value v_2, and $t - 1$ gems of value v_1; if ξ does not happen, the participant captures the gem of value v_2, and $t - c - 1$ gems of value v_1. Thus, the expected gain of the participant is equal to

$$
\begin{aligned}
&\Pr[\xi] \cdot (v_2 + (t-1) \cdot v_1) + (1 - \Pr[\xi]) \cdot (v_2 + (t-c-1) \cdot v_1) \\
&= v_2 + \frac{(t-1) \cdot (t-c-1)}{n} \cdot v_1 + \frac{(n-t+c+2) \cdot (t-c-1)}{n} \cdot v_1 \\
&= v_2 + \frac{t-c-1}{n} \cdot v_1 \cdot ((t-1) + (n-t+c+2)) \\
&= v_2 + \frac{(t-c-1) \cdot (n+c+1)}{n} \cdot v_1 \\
&= \frac{n}{\phi} + \frac{\left(\frac{n}{\phi} - \left(\frac{2}{\phi} - 1\right) \cdot n\right) \cdot \left(n + \left(\frac{2}{\phi} - 1\right) \cdot n\right) \pm O(n)}{n} \\
&= \frac{n}{\phi} + \frac{n^2 \cdot \left(-\frac{1}{\phi} + 1\right) \cdot \frac{2}{\phi} \pm O(n)}{n} \\
&= \frac{n}{\phi} + n \cdot \left(1 - \frac{1}{\phi}\right) \cdot \frac{2}{\phi} \pm O(1) = \frac{n}{\phi} + n \cdot (2 - \phi) \cdot \frac{2}{\phi} \pm O(1) \\
&= \frac{n}{\phi} + n \cdot \left(\frac{4}{\phi} - 2\right) \pm O(1) = \left(\frac{5}{\phi} - 2\right) \cdot n \pm O(1) = (5 \cdot \phi - 7) \cdot n \pm O(1).
\end{aligned}
$$
□

The final result of this section readily follows from the above bounds.

Corollary 6. *There exist games with* n *gems such that non-adaptive strategies can get no more than a fraction of*

$$\frac{2}{5^{3/2} - 9} + O\left(\frac{1}{n}\right) \approx 0.91728\ldots$$

of the optimal (adaptive) strategy's expected gain.

Proof. We apply Corollary 4 and Theorem 5, to conclude that the game of Definition 2 has a ratio between the expected gain of its best non-adaptive strategy, and the expected gain of its best adaptive strategy, not larger than

$$\frac{n + O(1)}{(5 \cdot \phi - 7) \cdot n - O(1)} = \frac{1}{5 \cdot \phi - 7} + O\left(\frac{1}{n}\right) = \frac{1}{5 \cdot \frac{\sqrt{5}+1}{2} - 7} + O\left(\frac{1}{n}\right)$$
$$= \frac{2}{5^{3/2} + 5 - 14} + O\left(\frac{1}{n}\right) = \frac{2}{5^{3/2} - 9} + O\left(\frac{1}{n}\right). \qquad \square$$
